# Clinically mild encephalitis/encephalopathy with a reversible splenial lesion of the corpus callosum in childhood: a single-center experience

**DOI:** 10.3906/sag-2110-73

**Published:** 2021-12-25

**Authors:** Halil ÇELİK, Betül Emine DERİNKUYU, Erhan AKSOY, Ülkühan ÖZTOPRAK, Nesrin CEYLAN, Ebru AZAPAĞASI, Suna ÖZDEM, Melek Melehat OĞUZ, Deniz YÜKSEL

**Affiliations:** 1 Department of Pediatric Neurology, University of Health Sciences, Dr. Sami Ulus Maternity and Children’s Health and Diseases Training and Research Hospital, Ankara, Turkey; 2 Department of Radiology, University of Health Sciences, Dr.Sami Ulus Maternity and Children’s Health and Diseases Training and Research Hospital, Ankara, Turkey; 3 Department of Pediatric Intensive Care, University of Health Sciences, Dr.Sami Ulus Maternity and Children’s Health and Diseases Training and Research Hospital, Ankara, Turkey; 4 Department of Pediatric Infectious Diseases, University of Health Sciences, Dr.Sami Ulus Maternity and Children’s Health and Diseases Training and Research Hospital, Ankara, Turkey; 5 Department of Pediatrics, University of Health Sciences, Dr.Sami Ulus Maternity and Children’s Health and Diseases Training and Research Hospital, Ankara, Turkey

**Keywords:** MERS, childhood, treatment, prognosis

## Abstract

**Background/aim:**

Mild encephalitis/encephalopathy with a reversible splenial lesion (MERS) is a rare clinicoradiological syndrome that typically presents with central nervous system symptoms such as loss of consciousness, seizure, headache, and ophthalmoparesis.

**Materials and methods:**

Here, we highlight the characteristics of this syndrome together with the clinical and MRI findings of 6 pediatric patients with MERS.

**Results:**

Between January 2017 and October 2020, 6 patients with MERS (3 boys and 3 girls) presented to our center. The mean age was 122 ± 54.6 (min-max: 44–180) months. None of the patients had a chronic disease. In our study, infectious agents were detected in 4 patients (66.6%), while noninfectious causes (one seizure and the other hyponatremia) were detected in two patients. All of our cases were discharged without any sequelae after an average of 12.1 ± 7 (min–max: 4–20) days of hospitalization. In 1 patient (case 6), control MRI could not be performed, and the radiological recovery of our other patients was shown to be between 14 days and 2 months.

**Conclusion:**

MERS is an acute encephalopathy with good prognosis and should be considered by neurologists in differential diagnosis due to its variable clinical presentation and specific MRI findings.

## 1. Introduction

Mild encephalitis/encephalopathy with a reversible splenial lesion (MERS) is a rare clinicoradiological syndrome that typically presents with central nervous system symptoms such as loss of consciousness, seizure, headache, and ophthalmoparesis. Although its pathogenesis is not fully understood, causes such as infectious agents (e.g., rotavirus, adenovirus, influenza A and B, dengue virus, mumps, *Mycoplasma pneumoniae*, *Streptococcus pneumoniae*), hyponatremia, and the use or discontinuation of antiepileptic drugs have been implicated in the literature. The prognosis is generally good, as clinical and imaging findings resolve completely within a few months [1–6].

The condition is subdivided into two types based on the extent of involvement. In MERS type 1, which is the more common type, lesions are limited to the corpus callosum splenium, while, in MERS type 2, the lesions involve the whole corpus callosum or spread symmetrically to the white matter. The absence of gadolinium enhancement in the white matter lesions, the presence of reversible diffusion restriction in MRI, and the hyperintense appearance in fluid-attenuated inversion recovery (FLAIR) and T2-weighted sequences suggest a process characterized by separation of the myelin layers due to edema and local infiltration of inflammatory cells [7]. Although most cases have been reported from the Asian continent, especially from Japan, cases have also been reported from various European nations, including our country [3,8]. Here, we highlight the characteristics of this syndrome together with the clinical and MRI findings of 6 pediatric patients with MERS.

## 2.Patients and methods

Six pediatric patients diagnosed with MERS according to the Takanashi [7] diagnostic criteria between January 2017 and September 2020 in the SBU Dr. Sami Ulus Gynecology, Obstetrics and Pediatrics Training and Research Hospital were included in the study. Patient data such as demographic characteristics, prodromal and neurological symptoms, neurological examination findings, MRI, and electroencephalogram (EEG) findings, laboratory results, treatment, and outcomes were reviewed. All MRI examinations included T2-weighted, FLAIR, T1-weighted, contrast-enhanced T1-weighted imaging, diffusion-weighted imaging (DWI), and apparent diffusion coefficient (ADC) maps. All MRI examinations were evaluated by an experienced pediatric radiologist. MRI and EEG were performed within 48 h of the onset of neurological symptoms (mean 24.8 ± 22.3 h for MRI and 22.6 ± 20.8 h for EEG).

## 3.Results

Between January 2017 and October 2020, 6 patients with MERS (3 boys and 3 girls) presented to our center. The mean age was 122 ± 54.6 (min-max: 44–180) months (Table). None of the patients had any chronic diseases.

### 3.1. Case 1

A 4-year-old girl with no previous complaints was admitted to the emergency department with fever for 2 days and transient vision loss that recurred several times during the day. General physical examination and vital signs were normal; hemogram and biochemical parameters showed no abnormalities.

**Figure 1:**
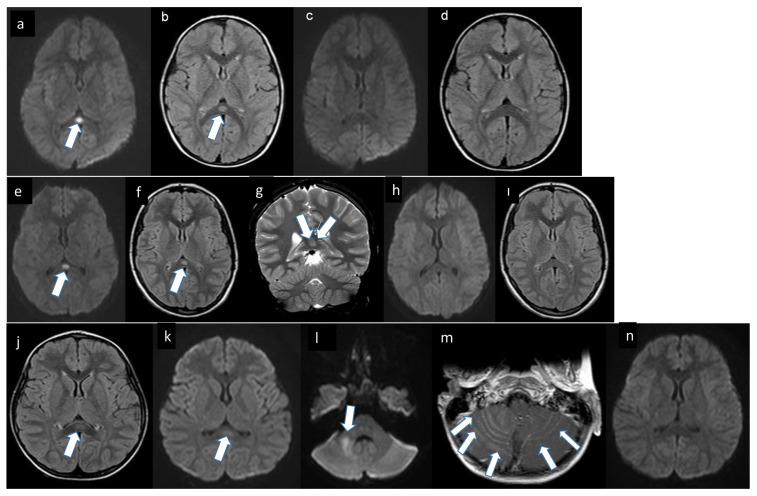
(a–n) legend**:** a: Diffusion-weighted image of the patient shows restricted diffusion in the splenium of the corpus callosum *(arrow)*. b: FLAIR image demonstrates high signal intensity in the splenium of the corpus callosum*(arrow)*. c,d:On follow-up MRI, the lesion in the splenium of corpus callosum was totally resolved. e: Diffusion-weighted image of the patient reveals restricted diffusion in the splenium of the corpus callosum *(arrow)*. f: Axial plane FLAIR and g: coronal plane T2 weighted images demonstrate high signal lesion in the splenium of the corpus callosum compatible with edema *(arrow)*. h,ı:On follow-up MRI, the lesion in the splenium of corpus callosum was totally gone. j: FLAIR image of brain MRI shows high signal intensity in the splenium of the corpus callosum *(arrow)*. k,l: Diffusion-weighted image shows restricted diffusion in the splenium of the corpus callosum and right side of the middle cerebellar peduncle. m: Post contrast T1 weighted image shows the leptomeningeal enhancement of the cerebellar folia more prominent on the right side than the left compatible with cerebellitis (arrows). n: On the follow-up MRI, the DWI image reveals the restricted diffusion in the splenium of corpus callosum was totally gone.

Detailed eye examination and EEG findings were normal. On lumbar puncture, cerebrospinal fluid (CSF) parameters were normal, and no microorganisms were detected in direct examination. Brain MRI revealed a hyperintense, diffusion-restricting oval lesion in the splenium of the corpus callosum in T2-weighted sequences (Figures 1a,1b). Infectious and metabolic evaluations revealed no factors that could explain this clinical and neuroradiological presentation. Cranial MRI obtained at 1-month follow-up showed that the lesion had resolved (Figures 1c,1d).

### 3.2. Case 2

A 12-year-old previously healthy girl presented with symptoms of upper respiratory tract infection for 3–4 days and sudden-onset strabismus with double vision. Vital signs were normal and physical examination was unremarkable except for the restricted movement of the left eye. Fundus examination was normal, and laboratory tests indicated that hemogram and biochemistry parameters were within reference ranges.

Brain MRI showed a diffusion-restricting millimetric lesion in the corpus callosum splenium that was hypointense in T1-weighted and hyperintense in T2-weighted sequences (Figures1e–1g). In etiological investigations, EEG was normal, CSF opening pressure on lumbar puncture was 58 cmH_2_O, CSF parameters were normal, and no microorganisms were detected on direct examination. Among infectious parameters, Epstein–Barr virus (EBV) capsid antigen antibodies (immunoglobulin [Ig] M and G) were positive, EBV nuclear antigen (EBNA) IgG antibodies were negative, and polymerase chain reaction (PCR) assay revealed an EBV load of 3.2×10^3^ copies/L. CSF PCR testing was negative for EBV and other pathogens. Based on these clinical, serological, and radiological results, she was diagnosed with MERS due to primary EBV infection. She was followed without treatment and brain MRI examination at the first month showed that the lesions had resolved (Figures 1h,1ı).

### 3.3 Case 3

A 5-year-old girl who had no known illnesses was referred to us due to prodromal symptoms such as diarrhea and fever for 2 days, transient generalized tonic clonic seizures that occurred 3 times in the same day, and sleepiness. At admission, her Glasgow Coma Scale (GCS) score was 13 (eye opening response: 4, verbal response: 4, motor response: 5) and other vital signs were normal except for a temperature of 38.2°C. She was slightly dehydrated and drowsy but there was no focal neurological deficit. Laboratory tests indicated normal hemogram and biochemical parameters. The first EEG was performed 1 day after admission and demonstrated diffuse slowing but no epileptic activity. On lumbar puncture, CSF protein and glucose levels were normal, and no microorganisms were detected in direct examination. Brain MRI revealed increased T2 signal intensity in the dentate nucleus and middle cerebellar peduncle in the right cerebellar hemisphere, intense contrast enhancement in the cerebellar foliage, diffusion restriction at the level of the dentate nucleus and middle cerebellar peduncle in DWI arrays, and hyperintense, diffusion-restricting oval lesions in the corpus callosum splenium in T2-weighted and FLAIR sequences (Figures 1j–1m). Rotavirus RNA was detected in stool by PCR but could not be evaluated in CSF. Encephalitis was suspected due to the presence of fever accompanied by drowsiness, and treatment with ceftriaxone and acyclovir was initiated. During follow-up, the patient’s drowsiness regressed, but she showed signs of ataxia and aphasia. Intravenous immunoglobulin (IVIg) was administered, but there was no clinical response. CSF viral PCR test and blood and CSF cultures were negative, and treatment with pulse methylprednisolone was initiated. Her general condition gradually improved, and she was discharged with no sequelae on day 16 of hospitalization. Brain MRI examination at 2 months showed that the lesions had resolved (Figure 1n).

### 3.4. Case 4

A 13-year-old boy was admitted to our emergency department with fever for 5 days, hypersomnia for 1 day, and an ataxic gait. It was learned that his family had coronavirus disease 2019 (COVID-19) 15 days earlier, and the patient had been in quarantine with contact isolation. A COVID-19 test was negative at that time. His body temperature was 39 °C, respiration rate was 30 breaths/min, oxygen saturation was 96% in room air, and blood pressure was 122/69 mmHg. He had transient agitation, excessive sleepiness, loss of orientation to place and time, and his GCS score was 13 (eye-opening response: 4, verbal response: 4, motor response: 5). Laboratory test results were as follows: hemoglobin 12.9 g/dL, leukocyte count 14100/μL, platelet count 73000/μL, serum sodium 135 mmol/L, alanine aminotransferase 345 U/L, aspartate aminotransferase 295 U/L, and C-reactive protein 342 mg/L. Asymmetric septal hypertrophy and myocarditis were detected on echocardiography.

**Figure 2:**
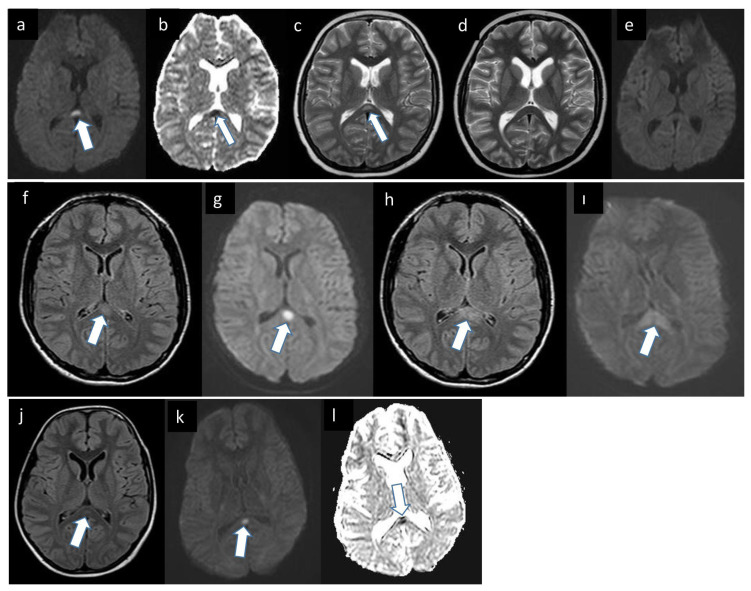
(a–l) legend: a: Diffusion-weighted image of the patient shows restricted diffusion in the splenium of the corpus callosum *(arrow)*. b: Note the low ADC value as a dark signal *(arrow)* in the ADC map. c: T2 weighted image demonstrates high signal lesion in the splenium of the corpus callosum compatible with edema *(arrow)*. d,e: On follow-up MRI, the lesion in the splenium of corpus callosum was totally gone. f: FLAIR image of Brain MRI shows high signal lesion in the splenium of the corpus callosum compatible with edema *(arrow)*. g: Diffusion-weighted image of the patient shows restricted diffusion in the splenium of the corpus callosum compatible with cytotoxic edema *(arrow)*. h,ı: On the follow-up MRI, the FLAIR and DWI images reveal the enlargement of edema in the splenium of corpus callosum (arrows). j: FLAIR image of the patient demonstrates high signal lesion in the splenium of the corpus callosum*(arrow)*. k: Diffusion-weighted image shows restricted diffusion in the splenium of the corpus callosum *(arrow)*. l: Note the low ADC values as a dark signal *(arrow)* in the ADC map regarding cytotoxic edema.

Real-time polymerase chain reaction (RT-PCR) of a nasopharyngeal swab sample remained negative for severe acute respiratory syndrome coronavirus 2 (SARS-CoV-2) but anti-SARS-CoV-2 IgM and IgG were detected in the patient’s serum. Brain MRI showed a hyperintense, diffusion-restricting lesion in the corpus callosum splenium in T2-weighted and FLAIR sequences (Figure 2 a,b,c), while EEG demonstrated diffuse slowing and no epileptic activity. Based on these findings, he was admitted to the pediatric intensive care unit (PICU) with a diagnosis of multisystem inflammatory syndrome in children (MIS-C). Treatment with IVIg and oral prednisone was initiated. His fever and other symptoms (transient agitation, excessive sleepiness, loss of orientation to place and time) started to improve on day 2, and he was discharged with no sequelae on day 14 of hospitalization. Brain MRI examinations performed 2 weeks later showed that the lesion had resolved (Figure 2 d,e).

### 3.5. Case 5

A 16-year-old boy with headache and fever for 4 days had been treated at another medical center for a presumed upper respiratory tract infection and was admitted to our emergency department when his symptoms worsened during follow-up. His temperature at admission to our center was 39.2 °C and other vital signs were normal. He exhibited neck stiffness on physical examination and his GCS score was 15. There were no abnormalities in hemogram and biochemical analyses. EEG showed diffuse slowing and no epileptic activity. On lumbar puncture, CSF protein and glucose levels were normal, and the white blood cell count was 40/mm^3^ on direct CSF examination. *Coxiella burnetti* IgG was detected at titers of 1/128 for phase I and 1/1024 for phase II, and the patient was diagnosed as having Q fever-associated meningoencephalitis. Brain MRI revealed a hyperintense, diffusion-restricting lesion in the splenium of the corpus callosum in T2-weighted and FLAIR sequences (Figure 2 f,g,h). The patient was treated with doxycycline, and his clinical condition gradually improved until discharge on day 20 of hospitalization. He had no sequelae, and no lesion was observed on brain MRI examination 2 weeks later (Figure 2ı).

### 3.6. Case 6

A 13-year-old boy presented to the emergency department with complaints of abdominal pain and vomiting for 4 days. His vital signs were normal, but right lower quadrant tenderness was noted on physical examination, and abdominal ultrasound findings were compatible with acute appendicitis. During preparation for surgery, he exhibited nonsensical speech, excessive sleepiness, and occasional agitation. Brain MRI showed a hyperintense, diffusion-restricting lesion in the corpus callosum splenium in T2-weighted and FLAIR sequences (Figure 2j–2l). There was diffuse slowing on EEG, and no epileptic activity was detected. In laboratory tests, serum sodium was 122 mmol/L, but other results were within the normal range. With intravenous fluid therapy, his clinical condition improved within 24 h, and an appendectomy was performed. He was discharged with no sequelae on day 16 of hospitalization. Control cranial MRI was not performed because the patient had no complaints during 3 months of follow-up.

## 4. Discussion

MERS is a generally benign condition characterized by mild central nervous system symptoms and a temporary lesion in the splenium of the corpus callosum on cranial MRI. It can occur after discontinuation of antiepileptic drugs, abrupt cessation of steroid therapy, hypoglycemia, and infections (especially viral upper respiratory tract infections such as influenza and EBV). Patients typically present with symptoms such as headache, confusion, ataxia, seizure, delirium, and coma [9–10]. Although factors such as cytotoxic edema, focal inflammatory changes, electrolyte changes in the cell membrane, and focal demyelination due to antiepileptic drugs have been implicated, the reason for the specific involvement of this region and its pathophysiological mechanisms have not been clarified [11,12]. In this study, we describe the clinical features and outcomes of 6 patients with MERS who presented to the neurology department of a tertiary health center. Prodromal symptoms in children are not specific, and although fever is the most common symptom, gastrointestinal and respiratory symptoms have also been reported [8,13]. In our study, 4 (66.6%) of our patients had prodromal symptoms in the form of fever (66.6%), 1 (16.6%) as diarrhea, and 1 (16.6%) as abdominal pain. In different case series, the most common neurological symptoms have been reported as a change in consciousness, behavioral change, ataxia, motor weakness, and seizures. In two separate studies from Japan, it was reported that 30% [13] and 50% (8) of pediatric cases had seizures. In addition, in another study conducted in Australia [14], it was found that 14.3% of children with MERS presented with seizures. Consistent with the literature, the neurological symptoms of our patients at admission included change in consciousness, headache, seizure, ataxia, and ophthalmoparesis.

Various infectious agents have been reported to be associated with MERS in children. In our study, infectious agents were detected in 4 patients (66.6%), while noninfectious causes were detected in two patients (seizure and hyponatremia).

One of our patients (case 3) developed febrile seizure, ataxia, and aphasia after rotavirus enteritis. Chen et al. [15] reported that 3 of 5 rotavirus-associated MERS patients had febrile seizures in their study. Rotavirus-related MERS or acute cerebellitis cases have been reported previously. Also, Takanashi J. et al. [16] reported that 6 of 11 rotavirus-associated cerebellitis patients had a splenial lesion in MRI. Similarly, in our study, we showed a spleen lesion on MRI in our patient with rotavirus-associated acute cerebellitis.

EBV-related MERS has been reported in older adult patients in the literature, but reports in children are less common [17,18]. In our study, we detected MERS associated with EBV infection in one of our patients (case 2). The patient presented with limited outward gaze and a CSF opening pressure of 58 cmH_2_O. This suggested that the ophthalmoparesis may have been due to increased intracranial pressure.

Although COVID-19 is a severe lower respiratory tract infection caused by SARS-CoV-2^1^, an immune-mediated syndrome called MIS-C has been identified in pediatric patients and can cause different neurological symptoms and splenial lesions on cranial imaging [19,20]. In our study, we detected MERS associated with SARS-CoV-2 infection in one of our patients (case 4). It should be kept in mind that children with COVID-19 infection may develop MERS. Another of our patients (case 5) had MERS associated with *C. burnetti*. As far as we know, MERS associated with *C. burnetti* has not been reported previously in the literature. Our case is remarkable in this respect.

Hyponatremia is another possible etiological factor in MERS. It has been suggested that hypotonic hyponatremia causes intramyelinic edema that results in transient reduced diffusion on MRI [21]. Hyponatremia rates reported in pediatric MERS vary greatly. Takanashi et al. [21] detected hyponatremia in 25 (84%) of 30 pediatric cases, while Zhang et al. [22] detected hyponatremia in 6 (24%) of 25 pediatric cases. We detected hyponatremia in one of our patients (case 6), who presented with acute appendicitis.

The most common EEG abnormality previously described in MERS cases is diffuse slow waves [3,7]. In our study, EEG was performed at a mean of 1.7 ± 0.5 (min-max: 1–2) days after admission and diffuse slowing was detected in 4 patients (66.6%), while the results of the other 2 patients (33.3%) were evaluated as normal. Our study was found to be compatible with the literature in this respect

Although there are no treatment guidelines for MERS, specific immunomodulatory therapy is not recommended because it has a generally good prognosis [3]. In our study, 3 patients (50%) improved clinically with treatment for the primary cause. Of the other 3 patients, one patient was treated with pulse methylprednisolone, IVIg, acyclovir, and antibiotic therapy due to the presence of acute cerebellitis with MERS; one patient was treated with prednisolone, IVIg, acyclovir, and antibiotic therapy due to the development of MIS-C secondary to SARS-CoV-2, and one patient was treated with IVIg, acyclovir, and antibiotic therapy due to encephalitis secondary to *C. burnetti*. All patients were discharged without sequelae after a mean of 12.1 ± 7 (min–max: 4–20) days of hospitalization. Control MRI was not performed in one patient (case 6), whereas the other patients demonstrated radiological recovery starting from the 14th day up to 2 months. However, the exact timing of complete normalization in MRI findings could not be determined because more frequent follow-up imaging is not available. In the literature, it has been reported that patients with MERS generally recover completely both clinically and radiologically [3,7].

The limitation of our study was that it was retrospective, and the number of patients is small compared to the large case series described previously. However, we believe these cases will contribute to the literature in terms of reporting infectious agents such as SARS CoV-2 and *C. burnetti* as infectious etiologies of MERS.

In conclusion, MERS is an acute encephalopathy with a good prognosis and should be considered by neurologists in differential diagnosis due to its variable clinical presentation and specific MRI findings.

## Figures and Tables

**Table:** The clinical findings in the pediatric patients presenting with reversible splenial lesion syndrome.

Patient no	Age (Mo) /sex	Preexisting illness	Etiology	Prodromal manifestations	Neurological manifestations	Na (mmol/ L)	CRP (mg/ dL)	WBC (10^9^ /L)	CSF examination cell count (10^6^/L)	Time to first MRI after the onset of symptoms	Lesion type	EEG findings	Treatment	Time to follow-up MRI after the onset of the symptoms	Hospitalstay interval (days)	Prognosis
**1**	44/F	No	Seizure	Fever	Seizure	137	<3	6400	Normal	2	I	Normal	None	30	4	CR
**2**	144/F	No	EBV	Vomiting	ophthalmaparesis	138	<3	8400	Normal	4	I	Normal	Acetozolamide	30	5	CR
**3**	64/F	No	RV	Vomiting Fever Diarrhea	Seizure/ ataxia/ mutism	135	<3	7800	Normal	3	I	Slow BA	MPT + IVIg Antibiotics, Acyclovir,	60	10	CR
**4**	156/M	No	SARS CoV-2	Fever	Headache/ drowsiness/ ataxia	139	342	14400	NE	2	I	Slow BA	Prednisolone + IVIg Antibiotics, Acyclovir,	14	14	CR
**5**	180/M	No	Coxiella	Fever	Headache / dizziness	140	64	8900	40	2	I	Slow BA	IVIg Antibiotics, Acyclovir,	30	20	CR
**6**	144/M	No	hyponatremia	Abdominal Pain Vomiting	drowsiness/ agitation	122	56	15400	NE	1	I	Slow BA	None	-	20	CR

M = male; F = female; Mo = Month, RV = Rotavirus, NE = not examined, BA = background activity, IVIg = intravenous immunoglobulin, MPT = Pulse methylprednisolone, CR = complete recover.

## Footnotes

1 World Health Organization. (2020). Coronavirus disease 2019 (COVID-19): situation report, 73. World Health Organization. Available at: https://apps.who.int/iris/handle/10665/331686
